# Neural Damage in Experimental *Trypanosoma brucei gambiense* Infection: The Suprachiasmatic Nucleus

**DOI:** 10.3389/fnana.2018.00006

**Published:** 2018-02-13

**Authors:** Chiara Tesoriero, Yuan-Zhong Xu, Dieudonné Mumba Ngoyi, Marina Bentivoglio

**Affiliations:** ^1^Department of Neuroscience, Biomedicine and Movement Sciences, University of Verona, Verona, Italy; ^2^Institut National de Recherche Biomedicale (INRB), Kinshasa, Democratic Republic of Congo; ^3^National Institute of Neuroscience (INN), Verona Unit, Verona, Italy

**Keywords:** human African trypanosomiasis, sleeping sickness, circadian rhythms, biological clock, astrocytes, neurodegeneration, arginine-vasopressin, vasoactive intestinal polypeptide

## Abstract

*Trypanosoma brucei* (*T. b.*) *gambiense* is the parasite subspecies responsible for most reported cases of human African trypanosomiasis (HAT) or sleeping sickness. This severe infection leads to characteristic disruption of the sleep-wake cycle, recalling attention on the circadian timing system. Most animal models of the disease have been hitherto based on infection of laboratory rodents with the *T. b. brucei* subspecies, which is not infectious to humans. In these animal models, functional, rather than structural, alterations of the master circadian pacemaker, the hypothalamic suprachiasmatic nucleus (SCN), have been reported. Information on the SCN after infection with the human pathogenic *T. b. gambiense* is instead lacking. The present study was aimed at the examination of the SCN after *T. b. gambiense* infection of a susceptible rodent, the multimammate mouse, *Mastomys natalensis*, compared with *T. b. brucei* infection of the same host species. The animals were examined at 4 and 8 weeks post-infection, when parasites (*T. b. gambiense* or *T. b. brucei*) were detected in the brain parenchyma, indicating that the disease was in the encephalitic stage. Neuron and astrocyte changes were examined with Nissl staining, immunophenotyping and quantitative analyses. Interestingly, significant neuronal loss (about 30% reduction) was documented in the SCN during the progression of *T. b. gambiense* infection. No significant neuronal density changes were found in the SCN of *T. b. brucei*-infected animals. Neuronal cell counts in the hippocampal dentate gyrus of *T. b. gambiense*-infected *M. natalensis* did not point out significant changes, indicating that no widespread neuron loss had occurred in the brain. Marked activation of astrocytes was detected in the SCN after both *T. b. gambiense* and *T. b. brucei* infections. Altogether the findings reveal that neurons of the biological clock are highly susceptible to the infection caused by human pathogenic African trypanosomes, which have the capacity to cause permanent partial damage of this structure.

## Introduction

Human African trypanosomiasis (HAT), also known as sleeping sickness, is a parasitic disease still threatening an estimated population of 65 million people in 36 countries of sub-Saharan Africa (WHO, 2017). The disease is caused by the extracellular protozoan parasites *Trypanosoma brucei* (*T. b.*), transmitted to humans through bites of tsetse flies (genus *Glossina*). After an epidemic in the 1990s, the number of reported cases has considerably declined due to sustained control programs, and the disease is targeted by WHO for elimination. About 2800 new cases of HAT have been recorded in 2015 (WHO, 2017). Concerns, however, are raised by underreporting and the identification of asymptomatic carriers (Franco et al., 2014; Büscher et al., 2017).

The disease occurs in humans in two forms: an acute form caused by *T. b. rhodesiense* and a chronic form caused by *T. b. gambiense*, both considered fatal if left untreated (Büscher et al., 2017). *T. b. gambiense* infections are currently responsible for 97% of reported HAT cases (WHO, 2017). The disease evolves in two stages (Kennedy, 2013; Büscher et al., 2017). The first, hemolymphatic stage, in which the blood and peripheral tissues are infected, progresses to a meningoencephalitic stage when trypanosomes invade the central nervous system. The infection leads insidiously to a complex neuropsychiatric syndrome including characteristic disturbances of the sleep-wake cycle with daytime somnolence and nocturnal insomnia, and alterations of the structure of sleep (Buguet et al., 2001, 2014), documented also in rodent models (Darsaud et al., 2003; Seke Etet et al., 2012; Laperchia et al., 2016, 2017). Most experimental studies on the brain in African trypanosomiasis have been based up to now, for obvious safety reasons, on the use of the *T. b. brucei* subspecies, which is a livestock pathogen not infectious to humans.

In mammals, the sleep-wake cycle represents a main endogenous biological rhythm driven by the suprachiasmatic nucleus (SCN), the master circadian pacemaker, located in the anterior ventral hypothalamus (van Esseveldt et al., 2000; Antle and Silver, 2005; Morin and Allen, 2006). Functional changes in the absence of overt structural alterations have been reported in the SCN of *T. b. brucei*-infected laboratory rats (Peng et al., 1994; Lundkvist et al., 1998, 2002). No studies, however, have been hitherto performed on the SCN of *T. b. gambiense*-infected hosts.

The aim of the present study was to examine the SCN after infection with the human pathogen *T. b. gambiense*. For this purpose, the multimammate mouse, *Mastomys natalensis*, was used as an animal model. *M. natalensis*, the most widespread rodent in sub-Saharan Africa (Coetzee, 1975), is a sensitive recipient of *T. b. gambiense* infection (Mehlitz, 1978; Büscher et al., 2005). Laboratory rats and mouse strains show little or no susceptibility to most *T. b. gambiense* isolates (Giroud et al., 2009), as also shown by early histopathological studies on the brain infection in mice, in which very few extravascular parasites were detected in the brain parenchyma (Van Marck et al., 1981), or the infection had a very long duration and succeeded only in a proportion of animals (Poltera et al., 1982). The SCN was also here investigated in *M. natalensis* infected with *T. b. brucei* for comparison with *T. b. gambiense* infection.

A wealth of data indicates that the SCN is composed anatomically and functionally by neuronal subpopulations on the basis of chemoarchitectonic criteria and circuitry organization (Van den Pol, 1980; Abrahamson and Moore, 2001; Antle and Silver, 2005; Morin and Allen, 2006; Moore, 2013; Hastings et al., 2014). Two neuropeptides which characterize main neuronal populations in the SCN are represented by vasoactive intestinal polypeptide (VIP) and arginine-vasopressin (AVP). VIP is expressed by neurons densely aggregated in the ventrolateral (VL) portion of the rodent SCN, which is also named “core” in the classical partitioning of the nucleus (Moore, 2013) and is the main target of retinal fibers in the SCN. AVP is mainly expressed by neurons located in the dorsomedial (DM or “shell”) portion of the nucleus, to which VIP neurons project in the intrinsic circuitry of the SCN and which gives origin to the SCN output. Communication between these two main subregions is effected by neural pathways and paracrine signaling (Hastings et al., 2014). Astrocytes are densely distributed in the SCN, where they represent a prominent and functionally important cell population (Morin et al., 1989; Becquet et al., 2008; Marpegan et al., 2011; Ng et al., 2011; Brancaccio et al., 2017), which can act as mediator of immune signals in the SCN (Leone et al., 2006). On this basis, the distribution of AVP and VIP neurons and astrocytes was here investigated in the SCN of *M. natalensis*, and neuronal density and astrocyte activation were evaluated after African trypanosome infection.

## Materials and Methods

### Infection and Tissue Processing

Adult *M. natalensis* of 3–4 or 5–6 months of age (lifespan in laboratory conditions: 13–15 months; Coetzee, 1975), both males and females, of 15–35 g body weight (for *T. b. brucei* infection) or 45–65 g body weight (for *T. b. gambiense* infection) at the beginning of the experiments were obtained by the local breeding colony, established in the animal facility of the Institut National de Recherche Biomedicale (INRB), Kinshasa, Democratic Republic of Congo (DRC). The animals were maintained under a 12 h:12 h light/dark cycle, with food and water *ad libitum*. Under approval of the ethical committee of the Ministry of Health of DRC, experimental procedures were performed in strict adherence to the European Communities Council (86/609/EEC) directives and the ARRIVE guidelines. All efforts were made to minimize animal number and suffering.

Two subspecies of trypanosomes were obtained from the cryobank collections of INRB: *T. b. gambiense* (originally isolated from a patient in DRC in 2006 and identified as *T. b. gambiense* MHOM/INRB/2006/11A), and *T. b. brucei* (AnTat 1.1E, a pleiomorphic clone isolated in 1966 from the blood of *Tralephagus scriptus* in Uganda). The cryostabilates were re-thawed in a water bath at 37°C and viability was assessed with the matching method (Herbert and Lumsden, 1976) before use. After dilution in 0.1 M phosphate buffer, pH 7.4, supplemented with glucose to achieve a concentration of 10^6.9^–10^7.2^ trypanosomes/ml, the inoculation was done by intraperitoneal injection of 0.25 ml per animal. The infected animals were monitored weekly. At each examination, blood samples were obtained from the tail tip to verify parasitaemia and body weight was recorded. Uninoculated animals, kept under the same conditions, were used as controls (*n* = 4 matched with either *T. b.* subspecies).

Previous disease monitoring at INRB has shown that *T. b. gambiense* infection of *M. natalensis* lasts about 4 months, and *T. b. brucei* infection about 2 months. On this basis, a survival time of 4 or 8 weeks was here adopted. At the time of sacrifice, no consistent body weight loss was found in the infected animals, which showed, however, high inter-individual variability. The animals (*n* = 3 or 4 per time point and parasite subspecies) were sacrificed, during daytime (*M. natalensis* is a nocturnal rodent; Coetzee, 1975), under anesthesia, by transcardial perfusion with ice-cold saline followed by freshly prepared 4% paraformaldehyde in PB. The brains were immediately removed, postfixed for a few hours, and stored until sectioning at 4°C in 0.01 M phosphate buffered-saline, pH 7.4 (PBS) containing 0.1% sodium azide. Following cryoprotection in 30% sucrose in PBS, the brains were sectioned coronally at a thickness of 30 μm with a freezing microtome. All sections through the SCN were collected in adjacent series of one every sixth section.

One series of sections from each animal was stained with cresyl violet for cytoarchitectural observation and cell counts. These sections were mounted onto gelatinized slides, air dried, and stained with 0.1% cresyl violet for 5–10 min. They were then dehydrated in a graded series of ascending ethanol concentrations, cleared with xylene, and coverslipped. The Nissl-stained series of sections was immediately adjacent to the series processed for the visualization of the neuropeptides AVP and VIP in the SCN with immunofluorescence (see below), in order to complement the cytoarchitectonic observations with chemoarchitectonic criteria.

### Immunocytochemistry

Neurons in the SCN were labeled using NeuN immunoperoxidase, as well as AVP and VIP immunofluorescence; glial fibrillary acidic protein (GFAP) was used as marker of astrocytes in immunoperoxidase and immunofluorescence. In all procedures, sections from control and infected brains were processed in parallel and in the same solutions. In preliminary experiments, attempts to stain microglia in sections from *M. natalensis* brains turned out to be unsuccessful, and microglia could not, therefore, be investigated.

For the study of neurons and astrocytes with immunoperoxidase, two series of sections from each animal were first preincubated in 1% H_2_O_2_ in PBS for 20 min to inactivate endogenous peroxidase activity, and then in a solution of 5% bovine serum albumin, 0.3% Triton X-100 in PBS for 1 h at room temperature. Subsequently, the sections were incubated overnight at 4°C with mouse monoclonal anti-NeuN antibodies (1:500, Chemicon, Temecula, CA, USA) or rabbit polyclonal anti-GFAP (1:500; Dako, Glostrup, Denmark) diluted in 0.2% Triton X-100 and 1% bovine serum albumin in PBS. After thorough rinsing, the sections were incubated for 2 h at room temperature in biotinylated secondary goat anti-rabbit or goat anti-mouse IgGs (1:200, Vector, Burlingame, CA, USA) in the solution used for primary antibody dilution. The sections were finally reacted with avidin-peroxidase complex (1:100, Vector) for 1 h and visualized using 3′,3-diaminobenzidine as chromogen. After rinsing in PBS, the sections were mounted onto gelatin-coated slides, air-dried, dehydrated, cleared and coverslipped. Specific immunostaining was absent in control sections in which the primary antibody was omitted.

Two adjacent series of sections from each animal were processed for double immunofluorescence to examine AVP or VIP together with GFAP. Briefly, following the blocking procedure in 5% normal donkey serum and 0.3% Triton X-100 in PBS, these sections were incubated overnight at 4°C in a mixture of mouse monoclonal anti-GFAP (1:500; Chemicon) antibodies, and rabbit polyclonal anti-AVP (1:1000; Phoenix, Burlingame, CA, USA) or anti-VIP (1:500, Santa Cruz Biotechnology, Santa Cruz, CA, USA) antibodies. Immunopositivity was visualized with species-specific secondary antibodies raised in donkey and conjugated with Cy2 or Cy3 (1:100; Jackson ImmunoResearch, Suffolk, UK).

Brain sections were also used to ascertain the presence of parasites in the parenchyma. These sections were processed for double immunofluorescence using a mixture of rabbit polyclonal antibodies anti-variant surface glycoprotein of the AnTat 1:1E stabilate (1:200; kindly provided by Philippe Büscher, Institute of Tropical Medicine, Antwerp, Belgium) to label trypanosomes, and goat polyclonal anti-glucose transporter-1 antibodies (1:100, Santa Cruz Biotechnology) to label blood vessel walls (Pardridge et al., 1990). Species-specific secondary antibodies were used as above.

The material processed for immunofluorescence was observed with a confocal laser scanning microscope (Zeiss, LSM 510) equipped with an argon laser emitting at 488 nm (Cy2) and a helium/neon laser emitting at 543 nm (Cy3).

### Data Analysis

All quantitative analyses were performed blindly of the experimental group assignment. Three animals per experimental group were used for all analyses.

#### Stereological Neuron Cell Counts

The borders of the SCN were delineated in each section using anatomical landmarks (optic chiasm and third ventricle) and cytoarchitectonic criteria. A previously validated stereological method (Abercrombie, 1946; Tsukahara et al., 2005) with modifications was used to estimate the number of neurons in either the whole SCN or its DM and VL subdivisions. Three equally spaced cresyl violet-stained or NeuN-immunostained sections through the rostrocaudal extent of the SCN were used. In cresyl-violet-stained sections, cell counts were performed in regions of interest (ROIs) randomly sampled using a 40× objective and a rectangular frame of 120 × 160 μm in the DM and VL portions (two ROIs per subregion per section, on each side of the SCN). The ROIs were consistently placed close to the ventral border of the SCN, near the optic chiasm and in the center of the dorsal portion of the SCN; the counting unit was a cell body containing a nucleus with a clearly visible nucleolus, discarding glial cells identified as smaller elements without nucleoli. In the NeuN-immunostained sections, the entire SCN area of dense immunoreactivity was used as ROI, and the counting unit was the immunoreactive cell nucleus, considering both faintly and intensely stained neurons. In each animal, the major axis of the neuronal nuclei was measured and averaged for the Abercrombie-based correction. According to this formula, the mean neuronal cell number per ROI was corrected by T/T+h, where T = section thickness (30 μm) and h = mean major axis of the neuronal nucleus (Liang et al., 2012).

The number of neurons was also counted in NeuN-immunostained sections through the hippocampal dentate gyrus (DG) of control and *T. b. gambiense*-infected *M. natalensis* at 8 weeks post-infection. This area was chosen on the basis of the key role of the hippocampus in the interaction between circadian rhythmicity and cognition (Kyriacou and Hastings, 2010), and the report of altered clock gene expression in the DG as long-term effect of acute sepsis (O’Callaghan et al., 2012). In each animal, three equally spaced sections through the middle portion of the hippocampus were sampled at corresponding rostrocaudal levels. Bilaterally, three ROIs (each of the same size used for cell counts in Nissl-stained sections of the SCN) were randomly sampled in the granule cell layer of the DG and the number of NeuN-immunostained neuronal nuclei was evaluated as above.

#### Densitometric Analysis of GFAP Immunosignal Intensity

For quantitative evaluation the GFAP immunoreactivity in the SCN, three equally spaced sections per animal through the middle portion of the SCN were used. The analysis was pursued at 4 weeks after *T. b. brucei* infection, as well as at 4 and 8 weeks after *T. b. gambiense* infection, vs. matched controls. Under constant calibrated parameters, images were taken using a digital camera (QImaging, Surrey, BC, Canada) connected to the microscope (objective 40×, NA 0.75). Analysis of digitized images was aided by the software Image Pro Plus 5.0 for Windows (Media Cybermetics, Silvers Springs, MD, USA). In each image derived from the DM and VL subregions, the values of GFAP immunosignal intensity were measured in a square ROI (100 × 100 μm). The mean optical density (OD) value from each ROI was normalized against the background, defined as the signal measured in an area devoid of specific immunostaining. Two ROIs per SCN subregion were evaluated in each section. The value was expressed as grand mean per SCN computed from the three mean values obtained from each section.

#### Statistics

Data are given as means ± standard error of the mean (SEM) in the entire SCN, and in the DM or VL portion, respectively. Neuronal density (mean number of neurons/ROI), relative neuronal density (neuronal density in infected animals vs. controls, setting controls as 1), and OD values of GFAP immunosignal were evaluated. Statistical evaluation was performed with the Student *t*-test or two way analysis of variance (ANOVA) followed by the Bonferroni’s *post hoc* test, as appropriate, in each series from *T. b. brucei* or *T. b. gambiense* experimental groups. Significance threshold was set at *P* < 0.05. The statistical analysis was performed using GraphPad.

## Results

### Trypanosome Neuroinvasion

**Figure 1:**
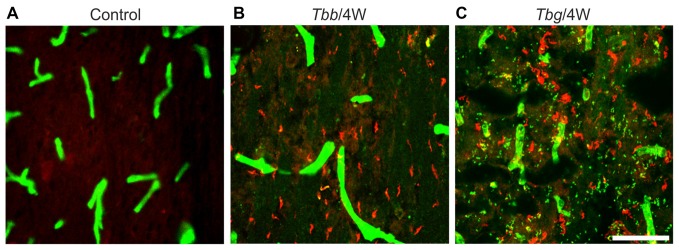
Confocal images showing *Trypanosoma brucei* (*T. b.*) parasites in the brain parenchyma of *Mastomys natalensis*. Parasites are visualized by variant surface glycoprotein immunostaining (in red), and blood vessels by anti-glucose transporter-1 immunostaining (in green). Control animals **(A)** were not infected. Note that at 4 weeks (4W) after infection with *T. b. brucei* (*Tbb*) **(B)** or *T. b. gambiense* (*Tbg*) **(C)** numerous parasites are found in the septum. Scale bar: 40 μm.

Numerous trypanosomes of either subspecies were observed in the brain of *M. natalensis* at 4 and 8 weeks post-infection (Figure 1). As demonstrated by the visualization of parasites and blood vessel walls in double immunofluorescence (Figure 1), many trypanosomes had invaded the parenchyma. *T. b. gambiense* parasites appeared more numerous than *T. b. brucei* in the host brain parenchyma, especially at 8 weeks post-infection, though no detailed analysis of parasite load was performed.

### The SCN of *M. natalensis*

**Figure 2:**
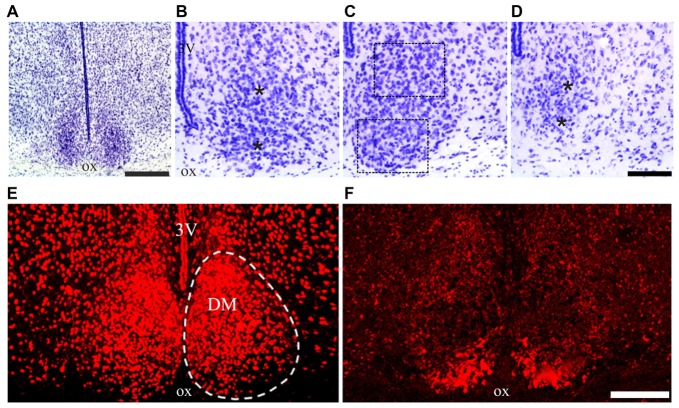
Suprachiasmatic nucleus (SCN) cytoarchitecture of *Mastomys natalensis*.** (A–D)** Cresyl violet-stained coronal sections through the rostral, middle and caudal levels of the SCN. The dotted lines in **(C)** outline the extent of the regions of interest (ROIs) used for the quantitative analyses; the asterisks in **(B,D)** are placed at the center of the same ROIs in the respective rostral and caudal sections. **(E,F)** Confocal microscopy images showing the distribution of the neuropeptide arginine-vasopressin (AVP) **(E)** and vasoactive intestinal polypeptide (VIP) **(F)**, in the middle level of the SCN. Abbreviations: DM, dorsomedial portion; ox, optic chiasm; VL, ventrolateral portion; 3V, third ventricle. Scale bars: 350 μm in **(A)**, 100 μm in **(B–D)**; 150 μm in **(E,F)**.

According to taxonomic criteria, the multimammate mouse *Mastomys* “holds an intermediate position between the house mouse and the ship (roof) rat” (quoted from Coetzee, 1975). In Nissl-stained sections, the cytoarchitectural features of the SCN of *M. natalensis* appeared similar to previous descriptions in the laboratory mouse and rat (Abrahamson and Moore, 2001; Moore et al., 2002). Located dorsal to the optic chiasm on either side of the third ventricle in the anterior hypothalamus, the SCN appeared as a compact, bilateral cell aggregates, with an oval shape in the coronal plane, especially in its middle third (Figures 2A–D). The boundaries of the SCN, clearly delimited ventrally by the optic chiasm, were distinguished also medially, laterally and dorsally on the basis of the high cell packing density with respect to the surrounding hypothalamic regions (Figure 2A).

Concerning the chemoarchitectural organization of the two peptidergic cell populations here examined, a relatively extended portion of the SCN, from its rostral pole to the caudal end, was filled by AVP-immunoreactive neurons, whose packing density decreased along the dorsoventral axis leaving unstained the most ventral portion of the nucleus (Figure 2E). VIP-immunopositive neurons were densely aggregated in a more restricted VL portion of the SCN (Figure 2F). This organization is grossly similar to that described in the laboratory mouse and rat (Abrahamson and Moore, 2001; Moore et al., 2002; Morin and Allen, 2006), although the dorsal portion of the SCN containing AVP neurons appeared relatively more extended in *M. natalensis* than in laboratory rodents.

### The SCN of *T. b.*-infected *M. natalensis*

**Figure 3:**
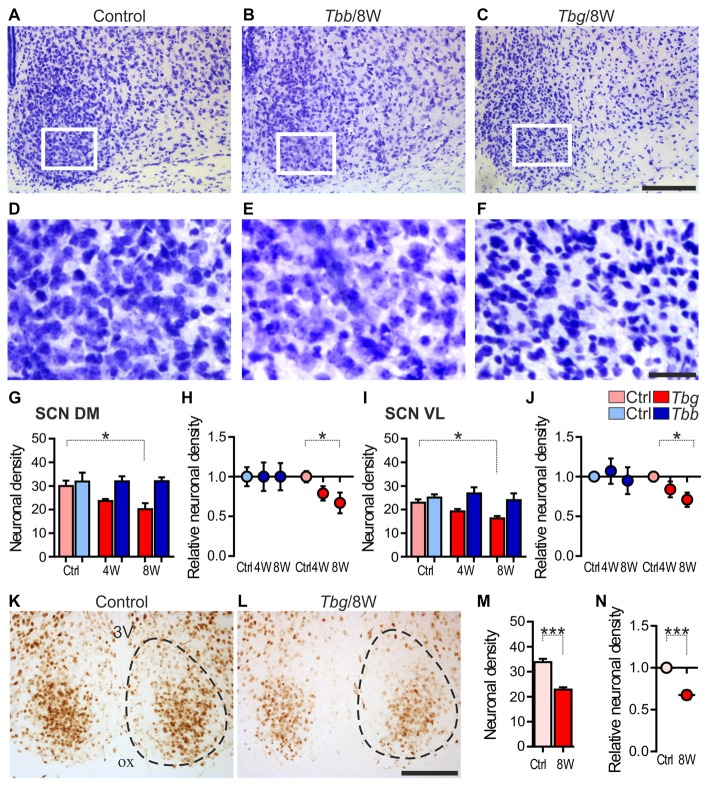
**(A–F)** Cresyl-violet-stained sections through the middle portion of the SCN of *Mastomys natalensis* at 8 weeks (8W) after infection with *Trypanosoma brucei* (*T. b*.) *brucei* (*Tbb*) or *T. b. gambiense* (*Tbg*) compared to uninfected controls **(A–F)** represent at higher magnification of the areas boxed in **(A–C)**, respectively. Note in **(F)** the features of neuronal rarefaction and gliosis. **(G–J)** Neuronal density (mean number of neurons ± standard error of the mean (SEM) per region of interest) in infected animals and matched controls (Ctrl), and relative neuronal density (in the infected animals vs. controls set as 1) evaluated in cresyl violet-stained sections in the DM and VL subregions of the SCN. ^*^P < 0.05 Bonferroni *post hoc* test following two-way analysis of variance (ANOVA). **(K–N)** NeuN immunostaining in the SCN and quantitative evaluations, as above. Cell counts were performed in the entire SCN area containing densely aggregated NeuN-immunoreactive neurons. The dotted line indicates the borders of the SCN, as derived from adjacent cresyl violet-stained sections. ^***^P < 0.001, Student t-test. Abbreviations: ox, optic chiasm; 3V: third ventricle. Scale bars: 100 μm in **(A–C,K,L)**; 25 μm in **(D–F)**.

In Nissl-stained sections through the SCN of the infected animals, the most striking change was a decrease of neuronal density and gliosis after *T. b. gambiense* infection, especially at 8 weeks (Figures 3C,F) as compared to matched controls (Figures 3A,D) and to *T. b. brucei*-infected animals (Figures 3B,E).

The neuronal cell counts in Nissl-stained sections confirmed the qualitative observations, showing about 17% neuronal loss (83 ± 9% neurons per ROI as compared to controls) in the SCN at 4 weeks after *T. b. gambiense* infection and about 30% neuronal loss (70 ± 11% neurons as compared to controls) at 8 weeks. The decrease of neuron density in the SCN of the *T. b. gambiense*-infected animals vs. matched controls was significant at 8 weeks (*P* < 0.05). No significant decrease of neuronal density as compared to controls was instead found in the SCN of *T. b. brucei*-infected animals.

Neuronal cell counts in the two subregions of the SCN showed that the neuronal density decrease documented after *T. b. gambiense* infection affected both the DM portion (79 ± 9% neurons per ROI as compared to controls at 4 weeks; 67 ± 13% at 8 weeks) and the VL portion (84 ± 10% neurons at 4 weeks; 71 ± 9% at 8 weeks), with a significant decrease in each SCN subdivision as compared to matched controls at the latest time point (Figures 3G–J).

In the NeuN-immunostained sections, the density of immunopositive neurons in the SCN appeared lower than in the cresyl violet-stained sections at corresponding levels, especially in the medial portions of the nucleus, and the immunostaining appeared fainter in the *T. b. gambiense*-infected animals than in controls, though clearly visible and with the same distribution as in controls (Figures 3K,L). This is consistent with the findings that the expression of NeuN, a reliable and conserved neuronal marker, can be downregulated in pathological conditions (Duan et al., 2016), and that in other rodent species (hamster and mouse) NeuN does not label all SCN neurons, in particular medially (Morin et al., 2011).

Regarding our quantitative evaluation, performed in the SCN regions of dense NeuN immunoreactivity, the density of immunopositive neurons at 8 weeks after *T. b. gambiense* infection showed a decrease (68 ± 5% NeuN-immunopositive neurons as compared to matched controls) similar to that documented in the cresyl violet-stained sections (see above), which was significantly lower than in the SCN of the control group (Figures 3M,N).

Regarding the observations of sections through the SCN processed for AVP or VIP immunofluorescence, the peptide immunosignal seemed to be downregulated and peptidergic cells appeared decreased in the *T. b. gambiense*-infected animals. This decrease, however, was not documented further by an objective quantitative evaluation.

**Figure 4:**
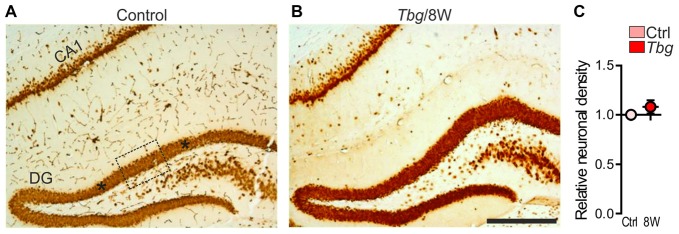
**(A,B)** NeuN immunostaining in the hippocampus at 8 weeks (8W) following *T. b. gambiense* (*Tbg*) infection and in a matched control. **(C)** Relative density (see the legend to Figure 3) of NeuN-immunopositive neurons in the dentate gyrus (DG) granule cell layer. The dotted lines in **(A)** outline the extent of one of the three ROIs used for the quantitative analyses, and the asterisks on each side are placed at the center of the other two ROIs. Scale bar: 250 μm.

In the hippocampus, NeuN immunostaining did not show differences in the distribution and labeling intensity between control and *T. b. gambiense*-infected animals (Figures 4A,B). At the quantitative evaluation, no significant changes were found at 8 weeks after *T. b. gambiense* infection animals vs. controls (Figure 4C).

**Figure 5:**
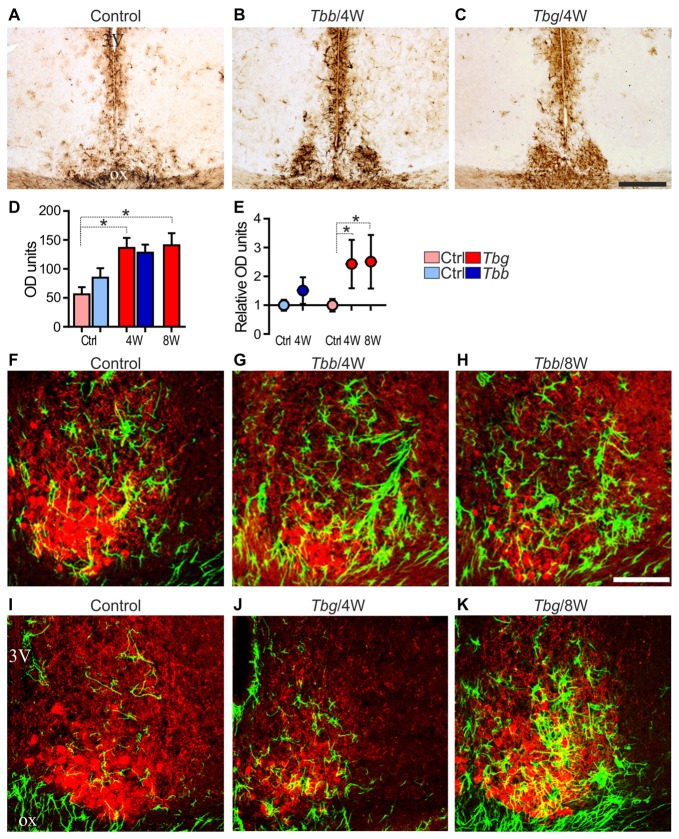
**(A–C)** Glial fibrillary acidic protein (GFAP) immunoreactivity of astrocytes in the mid-SCN of *M. natalensis*. Note the intense immunostaining in the SCN at 4 weeks (4W) after infection with *Trypanosoma brucei* (*T. b.*) *brucei* (*Tbb*) or *T. b. gambiense* (*Tbg*). **(D,E)** Quantitative data are presented as mean optical density (OD) values ± SEM per region of interest, and relative OD units evaluated in the infected animals vs. controls (Ctrl) set as 1. ^*^P < 0.05, Bonferroni *post hoc* test following two-way ANOVA. **(F–K)** Confocal microscopy merged images of GFAP immunofluorescence (in green) and VIP immunofluorescence (in red) in the mid-SCN. Note the features of marked astrocyte activation during the progression of the infection. Abbreviations: ox, optic chiasm; 3V, third ventricle. Scale bars: 250 μm in **(A–C)**, 100 μm in **(F–K)**.

As previously reported in laboratory rats (Tamada et al., 1998; Becquet et al., 2008), GFAP immunostaining in the SCN of *M. natalensis* was obviously more intense than in the immediately adjacent hypothalamic areas (Figure 5A). After trypanosome infection, GFAP immunostaining was markedly increased throughout the SCN, and especially in its VL portion (Figures 5B,C,F–K). The immunopositive astrocytes exhibited an activated phenotype, with hypertrophic and intensely immunostained cell bodies and processes, at both the sampled time points during the progression of *T. b. brucei* (Figures 5G,H) and *T. b. gambiense* (Figures 5J,K) infections.

Densitometric analysis demonstrated that the GFAP immunosignal intensity in the SCN was increased in *T. b. brucei*-infected animals at 4 weeks, although this trend did not reach statistical significance (Figures 5D,E). After *T. b. gambiense* infection, GFAP immunosignal intensity was significantly increased (a 2.5 fold increase) at both of the sampled time points.

## Discussion

This is the first histopathological investigation on brain structures in an animal model of infection with the human pathogen *T. b. gambiense* and is focused on the SCN. The main finding of the present study is the occurrence of structural damage in the master circadian pacemaker, with about 30% neuronal loss during the progression of the encephalitic stage of *T. b. gambiense* infection in *M. natalensis*. Despite the technical limitations in the use of NeuN as marker of SCN neurons (Morin et al., 2011) and the limited sample size, the finding was here replicated using cresyl violet staining and NeuN immunophenotyping. No neuronal loss was found in the same animals in the sampled hippocampal region, indicating that no widespread neurodegenerative phenomena had occurred in the brain. In the same recipient species, no significant neuronal loss was found in the SCN after *T. b. brucei* infection. Although it is likely that the small number of analyzed cases affected the statistical power, the comparison between the two infection paradigms suggests a structural vulnerability of the biological clock to *T. b. gambiense* infection.

Several studies in *T. b. brucei*-infected rodents and neuropathological studies in the brain of victims of *T. b. gambiense* HAT have indicated that African trypanosome infection leads to a neuroinflammatory pathology without the hallmarks of a widespread neurodegeneration (Quan et al., 1999; Kristensson et al., 2010; Buguet et al., 2014). A neuroinflammatory response to the infection was here confirmed by the activation of astrocytes in *M. natalensis* infected with *T. b. gambiense*, which was marked also after *T. b. brucei* infection, as previously observed after *T. b. brucei* infection in the laboratory mouse (Hunter et al., 1992; Keita et al., 1997) and rat (Kennedy, 2008).

No overt structural alterations have been observed in previous studies on the SCN of *T. b. brucei*-infected rats, as supported by the present data in *M. natalensis*. Functional alterations in the SCN of *T. b. brucei*-infected rats have, however, been reported, with changes in spontaneous and light-induced Fos expression (Bentivoglio et al., 1994; Peng et al., 1994), melatonin receptor binding (Kristensson et al., 1998), as well as reduced neuronal firing rate and phase advance of its peak (Lundkvist et al., 1998), and reduced excitatory synaptic activity (Lundkvist et al., 2002) in SCN slice preparations. Similar alterations of spontaneous single cell unit activity in SCN slices have been elicited by exposure to the proinflammatory cytokines tumor necrosis factor-α and interferon-γ (Lundkvist et al., 2002). This indicates that inflammatory mediators which are protagonist of African trypanosomiasis (Kristensson et al., 2010; Kennedy, 2013) can disrupt the synaptic machinery of SCN neurons (Lundkvist et al., 2002), which are cell autonomous oscillators (Hastings et al., 2014). In *T. b. brucei*-infected rats, a decrease in the expression of glutamate receptor subunits was also found, despite normal density and distribution of the excitatory retinal fibers which target the SCN (Lundkvist et al., 1998). The present experimental data sets indicate that the SCN could be even more susceptible to *T. b. gambiense* than to *T. b. brucei* infection, leading in this nucleus to widespread neuron loss.

Alterations of the daily alternation of sleep and wakefulness are a characteristic feature of HAT (Buguet et al., 2001, 2014). Other endogenous circadian rhythms, in particular some hormone secretion cycles, are also altered in HAT (Radomski et al., 1994; Brandenberger et al., 1996). The infection, therefore, affects the regulation of circadian rhythmicity. Experimental data based on *T. b. brucei* infection have indicated that the hypothalamus, and especially the posterior hypothalamus, is targeted early in the encephalitic stage by the active process of trypanosome traversal of the blood-brain barrier (Laperchia et al., 2016). The SCN, which expresses receptors to inflammatory mediators (Coogan and Wyse, 2008), could be especially sensitive to intrahypothalamic inflammatory signaling.

No information is available on the neuropathology of the SCN in the brain of HAT victims. Monitoring of the sleep-wake cycle in *T. b. gambiense* HAT patients with polysomnography during therapy has indicated that alterations of the sleep-wake cycle can slowly recover, which can be considered a clinical marker of therapy efficacy (Buguet et al., 1999; Mpandzou et al., 2011), as also shown by the use of non-invasive actigraphy (Njamnshi et al., 2012). Such clinical findings indicate, therefore, that SCN dysfunction in HAT can be largely compensated when the infection is cured.

Recovery after functional damage of the SCN has been reported in experimental paradigms of chronic (Palomba and Bentivoglio, 2008) and acute (O’Callaghan et al., 2012) neuroinflammation elicited by peripheral administration of the bacterial endotoxin, lipolysaccharide. Concerning structural damage, studies on partial SCN lesions with targeted methodological approaches, mostly based on genetic manipulations, have shown that lesions of cell populations with a specific chemical signature, e.g., those containing the calcium binding protein calbindin, which are located in the “core” of the rodent SCN (Antle and Silver, 2005), can lead to sustained loss of physiological and behavioral rhythmicity, while other microlesions can be compensated (Kriegsfeld et al., 2004). No detailed characterization of the SCN cell phenotypes spared by *T. b. gambiense* infection was possible in the present study. However, our findings indicate that neuronal loss was widely distributed in the SCN, therefore pointing to a neurodegenerative process which, based on experimental data (Kriegsfeld et al., 2004), could permit functional recovery. No clinical studies, however, are available on long-term, possibly subtle, sequels of permanent partial SCN damage in humans.

In conclusion, the present findings show that neurodegenerative events are caused in the SCN in a rodent model of infection with a causative agent of HAT. *T. b. gambiense* HAT is a chronic progressive disease with a mean duration of 3 years and, due to the nonspecific clinical signs and symptoms of the first stage, patients mostly present at the observation when the disease is already in the encephalitic stage (Büscher et al., 2017; Njamnshi et al., 2017), and the partial neuronal damage here documented in the SCN of an animal model might have already occurred. The outcome of an infection depends on the delicate and complex interplay between the pathogen and the host, and the potential translational implications of the present findings should, therefore, be regarded with caution. Even taking this into account, our study further underlines the urgency of an effective control and elimination of this dreadful African disease.

## Author Contributions

CT and Y-ZX: data analysis and manuscript preparation. Y-ZX tissue processing. DMN: infection design and animal experiments. MB: study design and article writing.

## Conflict of Interest Statement

The authors declare that the research was conducted in the absence of any commercial or financial relationships that could be construed as a potential conflict of interest.
